# Serious games as an educational strategy to control childhood obesity: a systematic literature review^1^

**DOI:** 10.1590/1518-8345.2509.3036

**Published:** 2018-09-03

**Authors:** Jéssica David Dias, Aline Natalia Domingues, Chris Mayara Tibes, Silvia Helena Zem-Mascarenhas, Luciana Mara Monti Fonseca

**Affiliations:** 2 Doctoral student, Escola de Enfermagem de Ribeirão Preto, Universidade de São Paulo, PAHO/WHO Collaborating Centre for Nursing Research Development, Ribeirão Preto, SP, Brazil.; 3 PhD, Associate Professor, Departamento de Enfermagem, Universidade Federal de São Carlos, São Carlos, SP, Brazil.; 4 PhD, Associate Professor, Escola de Enfermagem de Ribeirão Preto, Universidade de São Paulo, PAHO/WHO Collaborating Centre for Nursing Research Development, Ribeirão Preto, SP, Brazil.

**Keywords:** Child, Child Health, Pediatric Obesity, Video Games, Health Promotion, Health Education

## Abstract

**Objectives:**

to identify in the literature the efficacy of serious games to improve knowledge for and/or behavioral changes among overweight or obese children.

**Method:**

Systematic Literature Review. The Cochrane Systematic Reviews Handbook was used. The studies were collected from the following databases: Public Medline; Web Of Science; Science Direct; Latin American and Caribbean Health Sciences Literature; and the Health Game Research and Cumulative Index to Nursing & Allied Health Literature. The descriptors were video games and obesity, while the key word was serious games. Inclusion criteria were: studies classified as Randomized Clinical Trials written in English, Spanish or Portuguese and in which children were the subjects of the study.

**Results:**

2,722 studies were identified in the initial search and six studies remained in the final sample. The papers focused on encouraging behavioral changes in players, including physical exercise and improved eating habits. The studies report that serious games are a potential strategy to encourage positive coping with childhood obesity.

**Conclusion:**

research in this field is an expanding and promising strategy and serious games represent an alternative means to provide health education to children.

## Introduction

Childhood obesity is a complex condition that is related to genetic factors, nutritional intake, levels of physical activity, and environmental factors. According to the literature, being overweight is defined as Body Mass Index (BMI) between the 85^th^ and 97^th^ percentiles, while obesity is when BMI is equal to or above the 97^th^ percentile^1^.

Being overweight and obesity are social and epidemiological phenomena. Studies report that 42 million children under the age of five are already considered obese or overweight^2^ and this number is expected to increase to 70 million in 2025^3^.

Many intervention studies have addressed the problem of obesity and being overweight among children, which is already considered a public health problem^4^ ^-^ ^5^. Due to the intensive use of technology among the children of this generation, there are opportunities to promote health through technological devices, as these enable access to tools children are already familiar with^6^. In the last decade, various interventions have used technology to prevent obesity in school environments and in clinical practice, aiming to provide health education to children^7^ ^-^ ^8^.

Health education is seen as a strategy to improve the understanding of patients regarding a disease, enabling them to improve their general condition and decrease the use of health resources^9^. Individualized educational programs are efficacious but expensive^10^, while more traditional methods to provide education to patients, such as lectures or printed pamphlets, are more accessible but do not substantially improve clinical results^11^.

For this reason, innovative new systems of educational interventions that are friendlier to the target population have been created. These evidence-based interventions are intended to provide health education in a way that is more accessible to the public. One of the approaches is based on the use of games as a strategy to improve knowledge concerning health and a tool to complement medical treatment, therapies or disease management^12^.

With the increased popularity of video games over the last 30 years, researchers started exploring their potential for serious purposes^13^, the so-called “serious games”. These are defined as games implemented within an educational context, with specific learning objectives, which gamers are expected to achieve during game play^14^.

Digital game-based learning has the potential to spark interest among gamers, motivating them to engage in a task regularly over a long period of time, which is difficult to achieve with traditional learning material and approaches and, therefore, may make a difference in terms of educational efficacy^15^ ^-^ ^16^.

According to the literature, serious games in the health field can be innovative and potentially efficacious methods to improve knowledge, transmit a persuasive message, support behavioral change, and influence the results of health programs^17^. 

Therefore, this study’s objective was to identify in the literature the efficacy of serious games in improving the knowledge and/or behavioral change of overweight or obese children.

## Method

A systematic literature review was conducted using Preferred Reporting Items for Systematic Reviews and Meta-Analyses (PRISMA)^18^, addressing serious games as an educational intervention to cope with childhood obesity. 

A systematic review is intended to gather evidence available according to pre-specified eligibility criteria, with the goal to answer a specific question. Thus, a systematic method able to provide more reliable results is used, which leads to conclusions and supports decision-making^19^.

This systematic review was submitted to and approved by the international prospective register of systematic reviews - PROSPERO under No. CRD42016042272.

The Cochrane Systematic Review method^19^ was adopted and the stages proposed in the literature^20^ were followed, namely: establishment of a protocol; establishment of a research question; search for studies; selection of studies; critical assessment; data collection; and synthesis of data.

**Figure 1:**
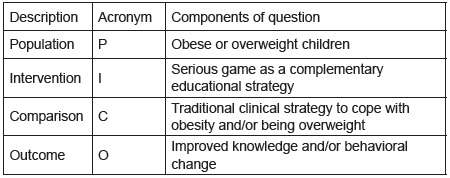
PICO framework of the research question

The PICO strategy, in which PICO stands for “Patient population/disease, Intervention or issue of interest, Comparison intervention or issue of interest, and Outcome”^21^ was used to establish the research question. Figure 1 presents the components of the research question according to PICO.

Therefore, the following research question guided this systematic review: “Does the use of a serious game as an educational strategy improve the knowledge and/or behavioral change of obese children when compared to the traditional clinical strategy?”

Traditional clinical strategy to control childhood obesity was defined as the follow-up and treatment provided by a regular pediatrician, including nutritional counseling and encouraging physical activity, and possibly involving other professionals, such as nutritionists an/or physical educators, but not necessarily^22^ ^-^ ^23^.

The papers were selected from the PUBMED, Web Of Science, Science Direct, LILACS, Health Game Research, and CINAHL databases. The terminology used here was based on the Medical Subject Headings (MeSH/PUBMED) and Health Science Descriptors, respectively video games and obesity, while the key word was serious games. 

The search strategy was initiated by first cross-referencing only the controlled descriptors and later including the non-controlled descriptors in the sequence in all the databases. The papers were then preselected by reading titles and abstracts and whenever doubts emerged concerning their content, these were separated to be later analyzed using their full texts. 

Inclusion criteria were: papers indexed in the aforementioned databases; written in English, Spanish or Portuguese; regardless of publication date; addressing children; and classified as Randomized Clinical Trials. The search occurred in January 2018 and secondary publications, such as review papers, books, monographs, theses and dissertations, were excluded. 

An instrument specific for analyzing Randomized Clinical Trials provided by the Consolidated Standards of Reporting Trials - CONSORT^24^ was used to verify whether the studies contained the information necessary to be included in the selection. The CONSORT instrument facilitates a critical interpretation of results because it enables researchers to extract details from the studies, as well as to verify what statistical analyses were conducted and how they were conducted^24^.

Two researchers independently selected the studies according to the stage of the research project. They first excluded studies by reading their titles (first stage), then by reading the abstracts (second stage), and finally by reading the full texts (third stage). An experienced third researcher was consulted in the case of disagreements or doubts.

The instrument was completed after reading the full text of each study, recording each stage in detail and verifying whether they provided what was necessary for a study to be considered a randomized clinical trial. 

After recording information with the analysis instrument, the data were organized in a Microsoft Excel worksheet. The papers were analyzed, after reading the full texts, to describe and classify the results and report the knowledge produced.

A total of 2,746 studies were identified in the initial search. All titles and abstracts were read and, after applying the inclusion criteria, six studies were selected. 

**Figure 2:**
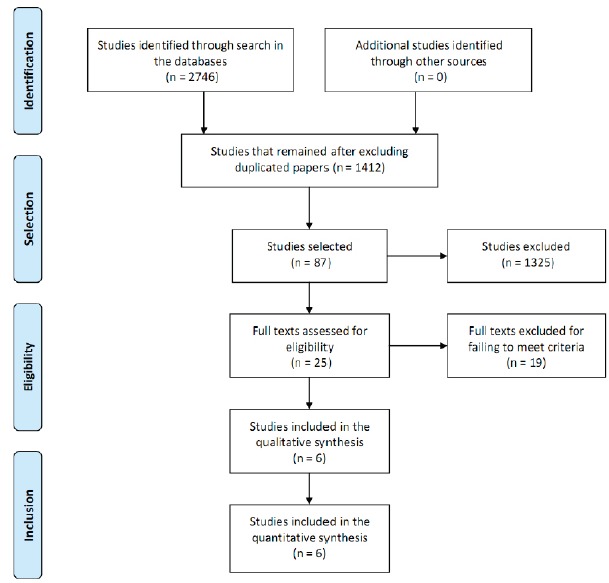
Synthesis of the selection process of studies according to the PRISMA model

A synthesis of the studies selection process following the PRISMA model is presented in Figure 2.

## Results

**Figure 3:**
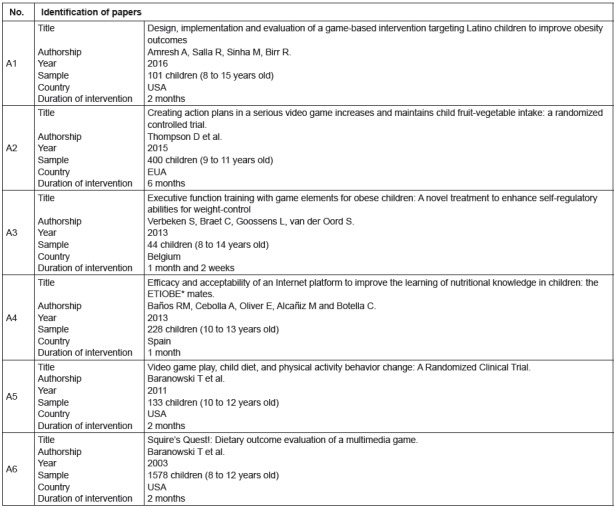
Studies selected for the review’s final sample

To better identify each study, the studies were organized in a sequence of letters and numbers, from A1 to A6 (Figure 3). 

*ETIOBE - e-therapy for obesity.

In regard to the year of publication, one study was found in each of the years 2003, 2011, 2015 and 2016, while two studies were found in 2013. In terms of country of origin, three clinical trials were conducted in the United States, one in Spain, and one in Belgium. The samples ranged from 1,578 individuals (largest sample) to 44 individuals (smallest sample). The children, public addressed by the studies, were aged between eight and 15 years old.

The first study analyzed^25^ ^)^ addressed a sample of 101 children, who were randomly assigned: 51 to the control group and 50 to the intervention group. The study focused on the use of serious games to improve knowledge concerning healthy nutrition and the benefits of adhering to good eating habits and daily exercise to prevent obesity. Latino children aged from eight to 15 years old were recruited for this study. 

The Intervention group played a serious game under a research assistant’s observation. The Control group received printed pamphlets addressing diet and exercise written both in English and Spanish. The study involved letting children play freely online at home while accompanied by a responsible adult. The researchers followed up with the participants via telephone to clarify potential doubts or address access problems; however, despite the presence of all these facilities, few individuals completed the game.

Most participants responded to a satisfaction survey stating they liked the game and would like to play it again in the future. The authors believe that using the game in clinical care with proper professional monitoring can motivate families to be more involved with the program designed to cope with obesity^25^.

The second study^26^ ^)^ reports the results of an assessment of a serious online game that presents fruits and vegetables in various ways to children as an additional health promotion strategy. The results concerning the intake of fruits and vegetables were assessed in the short and long terms.

The assessment included four randomized groups of school-aged children (nine to 11 years old) at three points in time: baseline; P1 (three months post-intervention); and P2 (three months post the last assessment). All the four groups played 10 episodes of the online serious game Squire’s Quest! II. The groups varied in how the intervention was implemented and were called: Action, Coping, Both and None. A goal was established for fruits and vegetable intake for the Action group and an action plan specifying how they would achieve this goal was also established. A goal was also established for the Coping group to eat more fruits and vegetables together with a coping plan, which identified potential barriers that could prevent them from achieving the goal. The Both group followed both plans of action and the last group, None, did not follow a plan at all.

Four hundred child-responsible adult pairs were recruited. A significant response of greater consumption of fruits, vegetables and greens was found (p <0.001) in one group only, the Action group, which showed a significant increase in the consumption of fruits and vegetables in P1 (p <0.0001) and P2 (p <0.0001). None of the other groups showed significant interactions. The authors concluded that establishing a plan of action might be an important component of successful interventions to increase and maintain the intake of fruits and vegetables in pre-adolescents. Video games intended to promote healthy eating habits are an efficacious intervention to promote behavioral change among children.

The third study^27^ conducted a randomized clinical trial in Belgium. All overweight children in the final phase of a 10-month hospital treatment program conducted in a pediatric medical center were invited to participate. The intervention was a training program incorporated into a gamified context. The serious game they developed was called “Brain game Brian”, that is, the name of the game’s main character was “Brian”. The control group maintained the routine care provided in the unit to fight obesity.

The children accessed the computer game at the clinic after school hours. The game consisted of 25 training sessions of approximately 40 minutes; that is, each session contained two blocs of tasks (of approximately 20 minutes each). The children trained four times a week (on fixed days) for a period of six weeks and were not allowed to play more than one 40-minute session per day. To motivate the children, each complete bloc of training tasks resulted in the development of a world in the game or extra power for the main character, Brian. With his extra powers, Brian created interventions to help people in his village, which resulted in happy villagers. A research assistant watched all the game sessions and clarified any doubts. 

The authors verified that the children in the intervention group showed significant improvement when compared to those in the control group. They were also more capable of maintaining their weight loss up to eight weeks after the training. This study shows promising evidence of the efficacy of a training program using serious games as a complementary strategy for obese children^27^.

The objective of the fourth study^28^ was twofold, to analyze the efficacy of the Etiobe Mates platform, which contains a number of serious games used as a tool to improve learning of nutritional processes among children, and identify the accessibility and playability of the games available on this platform.

After the parents consented, the researchers visited four schools to explain the program and 228 children (aged from 10 to 13 years old) were randomized and included in the study. The Intervention group was composed of 73 children and the Control group of 115. In the first meeting, the participants completed pretest questionnaires. The Control group received leaflets containing the same nutritional content as that provided by the ETIOBE Mates, only in a paper version. The group was then instructed to read the leaflet as many times as they wanted. The Experimental group, in turn, received a login and password to access the ETIOBE Mates, which included various sorts of nutritional content and healthy habits provided in the form of serious games. 

The Intervention group was instructed to navigate the platform and use everything freely for a period of two weeks. After two weeks, they completed the questionnaires once again (posttest) and reported on the ETIOBE Mates’ accessibility and playability. At the end of the study, the Control group also received logins and passwords to access the ETIOBE Mates to play the games.

Both groups increased their scores concerning nutritional knowledge; however, interaction of the experimental group was statistically significant, indicating superior acquisition of nutritional knowledge. The children considered the serious games platform to be a useful means to improve their nutritional knowledge. At the end of the study, the authors state that the online serious games can be an efficacious method to improve knowledge to prevent and treat diseases as information is presented in a different way to children^28^.

The fifth study^29^ ^)^ assessed the results of two serious games “Escape from Diab” and “Nano Swarm” in regard to children’s eating habits, physical exercise and adiposity. This randomized clinical trail was conducted with 153 children aged from 10 to 12 years old (103 in the Intervention group and 50 in Control group). The children were mainly recruited using radio commercials directed to African-American and Hispanic children living in Houston, TX, USA. One bilingual recruitment expert performed the preliminary screening. A raffle was drawn from 2008 to 2009 to select the participants for the two groups. The Intervention group played two serious games: “Escape from Diab” and “Nano Swarm”.

The assessments were performed before and immediately after the participants played the serious game. There were nine sessions and each lasted a minimum of 40 minutes. Each participant in the intervention group borrowed a computer with the games already installed. The coordinators of the intervention monitored the progress of the gamers through emails that were automatically sent whenever a session was concluded and also answered questions that emerged over the course of the intervention. The Control group did not play any serious game but received an activity available on the Internet that was composed of two parts, each including eight sessions in varied games focused on health. This experience was offered to the Control group to meet expectations of the participants to play video games related to health and avoid participant dropout. The immersion questions were applied to the Intervention group only; the Control group was excluded from the analysis of this subject.

The main outcomes measures were: fruit, vegetables and water portions and minutes of moderate to vigorous physical exercise. The participants were assessed on three non-consecutive days using a 24-hour dietary recall; on five consecutive days concerning physical activity (using accelerometers); and had their weight, height, waist circumference and triceps skinfold recorded. The results show that the children who played “Escape from Diab” increased their intake of fruits and vegetables by approximately 0.67 portions a day (p = 0.018), though the intake of water, frequency of moderate to vigorous physical activity, and body composition was unaltered. The authors concluded that the serious games promoted an increase in the intake of fruits and vegetables but further research is needed to determine components of games are ideal to maximize behavioral change among children^29^.

The sixth and last study^30^ reports the application of a serious game as an intervention implemented in 26 primary schools in the city of Houston, in the United States. The intervention was composed of Squire’s Quest!, a multi-media psycho-educational game that presents fruits and vegetables to children in various ways in the game.

The children were randomly assigned to the Intervention and Control groups. The Intervention group played ten sessions of Squire’s Quest!. The serious game was applied over the course of two months and each session lasted 25 minutes. The Control group did not receive any intervention; that is, it only completed the instruments that were necessary for later comparing the variables between the groups. Data from the 1,578 children were collected immediately before and after the program.

Four days of dietary intake were assessed before and after the intervention. The researchers used the Food Intake Recording Software System (FIRSSt), an instrument used to directly assess the 24-hour dietary intake of children. The categorical variables between the Intervention and Control groups were compared using the Chi-square test. The differences between groups concerning the intake of average portions of fruits and vegetables were tested using Student’s t test. 

The authors report that the children who participated in the intervention with Squire’s Quest! increased their consumption of fruits and vegetables by more than 1% compared to the children who did not receive the program. The researchers concluded that psycho-educational games have the potential to substantially change eating behavior. 

## Discussion

Based on the studies selected and analyzed in this review, the conclusion is that serious games used as a strategy to cope with obesity is an expanding field and its application has promising results that should not be ignored. It is, however, an incipient field and the limited number of randomized clinical trials that met this review’s inclusion criteria do not allow the efficacy of these games as an intervention to be determined.

Note that, even though there was an insufficient number of clinical trials to state that the use of serious games is an effective strategy of treatment, all the six studies included here indicate their participants were satisfied; three studies report significant results concerning the changing of eating habits; one reports the improvement of knowledge; and one study reports decreased body weight.

In regard to the serious game used as intervention, most authors developed their own game, validated it and later applied it to the target-population^25^ ^-^ ^28^ ^,^ ^30^; only one author used commercial games related to the theme that had been previously developed^29^.

There were differences regarding the duration of the interventions, which ranged from one to six months. The greater the time of exposure to and application of serious games, the more efficiently content was fixed and the greater was behavioral change among children.

The studies converged in various aspects, such as: children manifested great interest in the interventions and showed greater motivation because they were about digital games; significant improvement was found in terms of diet, the choice of healthier foods, behavioral change in the target-population, and greater levels of physical exercise; and improved knowledge concerning healthy eating. In general, the games were well-accepted by the children as a differentiated strategy to cope with obesity^26^ ^-^ ^29^.

Only one of the studies reports more controversial results^25^, mainly because some of the children did not complete the intervention as expected; in some cases, the game was interrupted. The authors did not clarify the reasons, though both the children and the parents assessed the game positively. This study was also the only one in which children were freely allowed to play the serious game online at home, without a researcher monitoring, which may have compromised the individuals’ continuing participation in the intervention.

The literature also corroborates the findings of the studies selected in this review: the use of serious games can positively aid coping with childhood obesity. These games are alternatives for providing health education to children, as this technology is flexible and can support education, as well as encourage more active learning^31^ ^-^ ^32^.

The introduction of serious games as an additional component in programs intended to enable patients to cope with disease can increase motivation and conformity to a program, improving the results of interventions^33^.

There is evidence in the literature that corroborates these findings. A meta-analysis identified 64 serious games that promote healthy life styles, revealing that the games had a statistically significant effect on behavior^34^. Other authors performed a systematic review with 19 studies addressing changes in health or safety behaviors among young individuals and verified that 17 of these papers reported at least one statistically significant effect concerning behavior^35^.

A systematic review analyzed 28 games intended to prevent childhood obesity utilized between 2005 and 2013 and reports that 40% of the studies obtained the desired effect on a variable related to adiposity^7^. Hence, substantial evidence supports the efficacy of serious games in improving knowledge and encouraging behavioral changes or even better health results.

Another point raised by this study’s review was the fact that the attention of a child is naturally captured by video games and the time children already spent playing can be used to promote health education^31^. It is interesting to develop games directed to decreasing obesity, for this generation of children is already accustomed to these technologies as entertainment. Such games can include educational content intended to increase their knowledge and self-care.

Additionally, even though the games identified in this review are inactive and sedentary in their natures, as they are played on tablets or computers, children can make better use of their time playing games available online using mobile devices in their daily routine.

Few systematic reviews identified in the literature examine the impact of technology-based interventions to fight childhood obesity. Some authors explored the effect of technological interventions, while others focused on the use of video games designed to prevent childhood obesity^7^ ^,^ ^36^. This review focuses on serious games, a more specific category of video games.

New studies are needed to include family members and the children’s responsible adults, something only one study addressed in this review did^25^. The parents are important for the adherence of children to interventions and the results they achieve, considering that parents are the role models for good eating habits, nutrition, and exercise, as well as controlling the environment and experiences of children^37^. Using digital games as a tool to direct or involve parents can also have a good cost-benefit relationship. Parents can be the target-population giving them access to games and tasks they are supposed to do by themselves, together with their children, or parallel with them.

Additionally, health education linked to schools is also a good alternative. Schools would benefit from incorporating serious games into their curricula, making it more attractive to students and approximating children to technology on their daily routine^38^. 

To achieve such results, however, more studies addressing the theme need to be analyzed in order to acquire greater understanding and determine the most appropriate and effective application of serious games in prevention and treatment programs directed to childhood obesity.

The results were heterogeneous in regard to the measures and assessment methods used. The analysis of games proposed in this study provides a structure for the organization of the diverse results and the impact serious games may have on children, but are also evidence of the persistent difficulties associated with the assessment of learning results or behavioral changes. 

These findings indicate that serious games encourage behavioral changes and improve knowledge among children; however, there is a need to standardize how these games are assessed. Even though improved knowledge and positive behavioral changes were found among children, more clinical trials are needed to acquire increasingly accurate evidence of these games’ efficacy.

## Conclusions

The results presented by this systematic review show there is interest and investment in the development and use of serious games to improve the knowledge and behavioral change of obese or overweight children. When the potential and popularization of serious games in the health field is considered, we can verify that research in this field is an expanding and promising strategy.

The games selected in this review focused on encouraging behavioral changes and improved physical exercise and eating habits among gamers.

It is worth noting that most studies reported the development of a game they presented to the children already in its functional version, focusing on games used in the clinical practice or applying them as an educational intervention. Only one author used well-disseminated commercial games, the objective of which is to be used within programs fighting obesity and promoting health. 

The results concerning the use of games as a strategy to cope with obesity reveal children accepted them well and that these games are seen as a potential motivator to maintain the adherence of children to interventions. Only one study reports controversial results. Even though the target audience was satisfied with its game, many of the individuals did not complete the intervention.

This review’s limitations included the fact that a meta-analysis was not possible because the studies addressed were heterogeneous and the fact that randomized clinical trials were chosen to control for common biases of experimental research, such as selection and confounding factors. Non-randomized controlled trials could have been selected instead, increasing the number of studies in this review. This option, however, would render the estimates less valid, considering that interventions with more questionable internal validity would have been included.
